# Fungal necrotizing otitis externa; challenges in diagnosis and therapy

**DOI:** 10.1007/s00405-025-09685-2

**Published:** 2025-10-03

**Authors:** Gaelle Vofo, Ofir Zavdy, Rona Bourla, Hila Elinav, Maya Korem, Sarah Israel, Reut Book, Fatima Azhraa Haddad, Dvir Bar-On, Tomer Menzely, Ohad Hilly, Sagit Stern Shavit

**Affiliations:** 1 Department of Otolaryngology- Head and Neck Surgery, Hadassah Medical Center, Jerusalem, 91120 Israel; 2 Department of Otolaryngology, Head & Neck Surgery, Rabin Medical Centers, Petah Tikva, Israel; 3 Department of Clinical Microbiology and Infectious Diseases, Hadassah Medical Center, Jerusalem, Israel; 4 Hebrew University School of Medicine, Jerusalem, Israel; 5 Gray faculty of medical and health sciences, Tel-Aviv University, Tel-Aviv, Israel

**Keywords:** Fungal culture, Fungal necrotizing otitis externa, Necrotizing otitis externa, Patient factors, Persistence/Recurrence, Systemic antifungal medication

## Abstract

**Purpose:**

Necrotizing otitis externa (NOE) is a severe infection with potential skull base involvement. The role of fungal pathogens and the need for antifungal treatment remain controversial. We aimed to evaluate clinical characteristics, diagnostic challenges, and microbiology results in fungal NOE and assess considerations for antifungal therapy.

**Methods:**

This retrospective cohort study was conducted at two tertiary centers. NOE patients, based on clinical and radiological findings, were retrospectively reviewed. Clinical characteristics, microbiological findings, and disease persistence were compared between patients categorized by antifungal treatment status: Combined antifungal-antibiotic therapy versus antibiotic therapy alone.

**Results:**

Of 125 patients, 25 received combined treatment (13 as initial treatment and 12 after persistent disease) and 100 received antibiotics alone. Combined treatment patients were younger, had prolonged symptoms, and more cranial nerve palsy, requiring longer treatment for persistent and aggressive disease. Fungi were found in 72% of combined-treatment cultures, but most were deemed unreliable by infectious diseases specialists, inconsistently leading to antifungal therapy.

**Conclusion:**

Antifungal therapy decisions in NOE lack standardization. We propose a protocol incorporating clinical and microbiological data to guide antifungal therapy. Prospective studies are needed to validate this protocol and establish standardized guidelines for fungal NOE treatment.

## Introduction

Necrotizing Otitis Externa (NOE), previously known as “malignant otitis externa,” is a severe, uncommon infection of the external auditory canal (EAC) that can extend into the temporal bone. NOE predominantly affects elderly [1], diabetic, and immunosuppressed patients [2], and its hallmark, temporal bone osteomyelitis, can be life-threatening, particularly when extending into the skull base [3, 4]. A high index of suspicion is necessary for prompt diagnosis [5], which typically relies on a comprehensive clinical evaluation, including a thorough medical history, meticulous physical examination, microbiological analysis, and imaging studies [6]. 

The role of fungi in NOE pathogenesis remains controversial. Although *Aspergillus fumigatus* was first reported as a causative agent in 1985 [7], subsequent case-series studies have indicated fungal involvement in 2–20% of NOE cases [8–13], particularly following prolonged antibiotic use [9, 10]. The four predominant fungal species include *Aspergillus fumigatus*, *Aspergillus flavus*, *Aspergillus niger*, and *Candida species* [7, 10, 13, 14]. Patients with fungal NOE often experience longer hospital stays, more complications, and require complex treatment regimens, including prolonged systemic antifungal agents (e.g., amphotericin B, voriconazole, fluconazole, posaconazole, itraconazole) in addition to antibiotics, surgical debridement, and management of underlying comorbidities [15]. Despite these challenges, survival rates of fungal and bacterial NOE appear comparable [10]. However, diagnosing fungal infection in NOE can be challenging. Fungal pathogens may not be identified in initial cultures, and their presence may be mistakenly attributed to contamination. Furthermore, the potential for significant toxicities associated with systemic antifungal medications can deter prompt treatment initiation [15–17]. This study aims to investigate the role of fungal infections in NOE by exploring the patient factors that influence the use of systemic antifungal medications and their impact on disease progression compared to patients treated solely for bacterial infections.

## Methods

A retrospective review was conducted of all cases diagnosed and treated for NOE or malignant otitis externa at two tertiary referral hospitals from January 1990 to December 2023. The respective hospitals’ ethical committees approved the research with a waiver of informed consent (0590-21-HMO and 0019-17-RMC).

### Patients

Patients were included if NOE was diagnosed based on all the following criteria: (1) signs and symptoms compatible with external otitis including severe otalgia, edema of the external ear canal, otorrhea, and granulations; (2) Failure to respond to anti-pseudomonas systemic and local treatment for at least one week; (3) Positive findings in one or more of the following imaging scans: evidence of bone erosions in the ear canal or adjacent skull bone on a CT scan, signs of skull base involvement on an MRI, or a positive uptake in radionuclide scan, such as methylene diphosphonate (MDP)-technetium-99 m (Tc99m) bone scan, a Gallium (Ga67) scan, or positron emission tomography (PET)-CT;

(4) Histology sample compatible with inflammation to rule out malignancy in all patients. Patients were excluded if they did not meet the above criteria or if information was missing regarding their treatment or outcome data.

To evaluate the role of treatment in NOE, we excluded patients who did not achieve complete recovery due to disease-specific mortality and treatment complications, assuming that treatment influence could not be reliably assessed in these cases. These cases were still reported in the descriptive analysis of overall outcomes.

A persistent/recurrent disease was defined as one that requires additional treatment beyond 8 weeks or recurring within a few months of treatment cessation, based on the patient’s symptoms and follow-up imaging.

### Data collection

Demographic characteristics, culture results, treatment approaches (including antibiotics and antifungals), treatment durations, and patient outcomes were collected for all included cases. All patients received initial antibiotic treatment according to their culture results or an empiric systemic antifungal treatment. Systemic antifungal therapy was added to the treatment regimen of some patients based on individual clinical considerations and culture results.

For this analysis, patients who received combined antifungal and antibiotic therapy from the outset were compared to patients who received antibiotics alone. Patients who initially received antibiotics and subsequently required the addition of antifungal therapy (after 8 weeks) due to persistent or recurrent disease were included in the initial antibiotic group. To minimize misclassification bias due to treatment crossover, we conducted all analyses in two ways: first, classifying patients according to their initial treatment (antibiotics only vs. combination from outset), and second, based on whether antifungal therapy was received at any point during the disease course.

Two infectious diseases specialists (HE, MK) independently reviewed patient cultures to assess their reliability in suggesting fungal infection. Cultures were classified as: (1) definite or highly probable fungal NOE- isolation of a fungal pathogen from a biopsy or isolation of the same fungal pathogen from at least two swabs obtained on different occasions, with a compatible microbiological staining. (2) Possible fungal NOE- isolation of the same fungal pathogen from at least two swabs obtained on different occasions, without a compatible microbiological staining. (3) Low probability of fungal NOE- e.g., mixed fungal and bacterial isolation, or fungal isolation from a single swab. Discrepancies in scoring between the two specialists were resolved by a third specialist (SI).

### Data analysis

The data was analyzed using SAS software (SAS ONDEMAND FOR ACADEMICS, version 3.8, Enterprise Edition). Categorical variables are presented as frequencies and percentages, and continuous variables as means and standard deviations. The chi-square test and Fisher’s exact test were used to calculate associations between categorical variables. The quantitative variable was compared between two independent groups using the two-sample t-test. Cohen’s kappa coefficient was used to calculate interrater reliability. A multivariate logistic regression model was constructed to identify independent predictors of persistent/recurrent disease. All statistical tests were two-tailed, and a p-value of 5% or less was considered statistically significant.

## Results

**Fig. 1:**
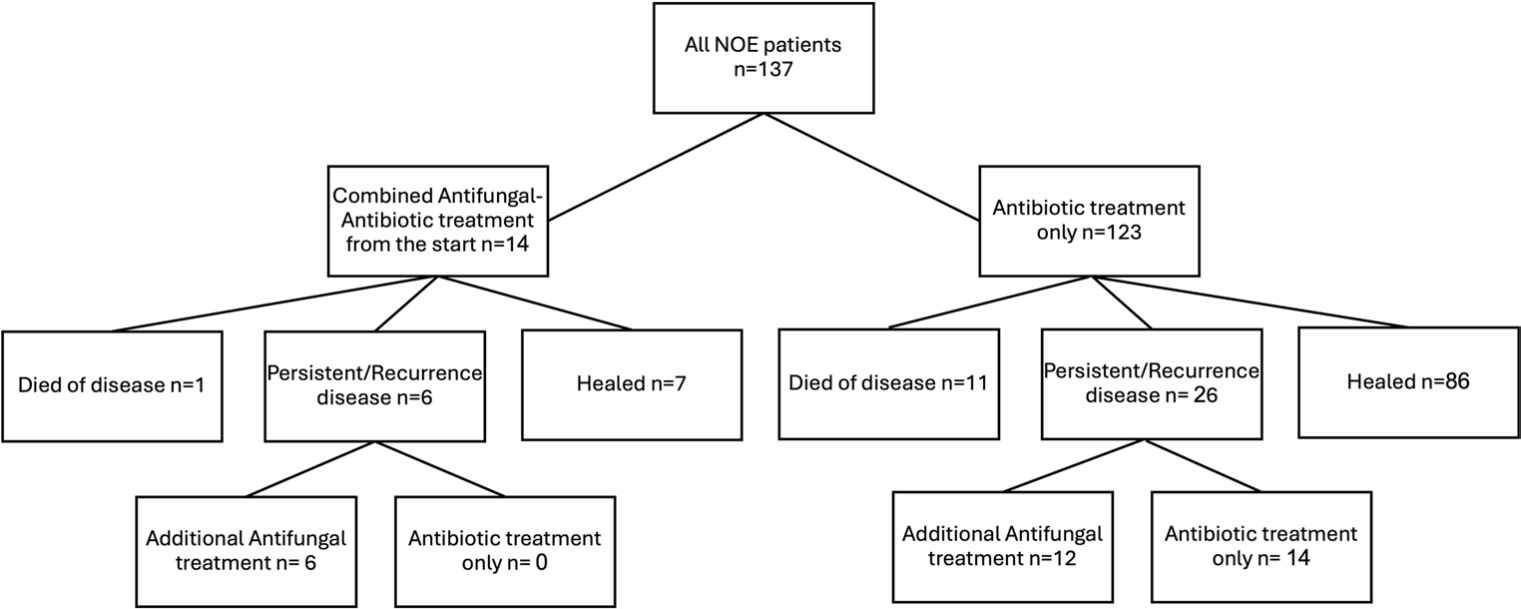
Distribution of the 137 cohort patients based on their treatment status and prognosis

One hundred and thirty-seven patients diagnosed with NOE met the inclusion criteria. Figure 1 illustrates the distribution of patients according to their treatment status and prognosis. In the general cohort, the mean age was 72.8 years (± 14.7), and 69.0% were male (*n* = 87). Diabetes mellitus was present in 79.2% (*n* = 99) of patients. The mean duration of hospitalization was 21.1 days (± 14), and the mean initial systemic antibiotic treatment duration was 5.49 weeks (± 2.3).

### Combined antifungal and antibiotic therapy from the outset vs. antibiotics alone

Of the 137 NOE patients 14 (10.2%) initiated a combined systemic antifungal and antibiotic treatment as the primary treatment protocol, while the others 123 patients (89.8%) received systemic antibiotics alone. One patient in the combined treatment group had died of disease (7%), six had persistent or recurrent disease (43%), and seven healed after their initial treatment course (50%). Eleven patients in the antibiotic-only treatment group had died of the disease (9%), 26 had persistent or recurrent disease (21%), and 86 healed after their initial treatment course (70%). Healing rate was higher in the antibiotic-only treatment group, however statistical significance was not reached (50% vs. 70%, *p* = 0.13).

Focusing on patients that healed after treatment, 100 patients recovered after one (*n* = 86) or two (*n* = 14) courses of antibiotic-only treatment (therefore assumed to have bacterial infection), while 25 patients healed after being treated with a combination of antifungal and antibacterial agents.

Of which 13 initiated a combined systemic antifungal and antibacterial treatment as the primary treatment protocol. Those treated with the combined protocol tended to be younger (66.4 (± 13.01) vs. 73.77 (± 13.27), *p* = 0.06), all had diabetes (100% vs. 76%, *p* = 0.045), had a long duration of symptoms (71.58 days (± 58) vs. 40.9 (± 39.8), *p* = 0.015) and had a higher rate of cranial nerve palsy (94% CN7, 6% CN6; 30.7% vs. 10.7%, *p* = 0.04) (Table 1).

**Table 1:** Sociodemographic and clinical characteristics of participants

Baseline characteristics n=125	Initial combined Antifungal-Antibiotic treatment n=13	Antibiotic treatment only n=112	P value^!^
**Age (mean +/- SD)**	66.4 +/-13.01	73.77 +/-13.27	0.061^^^
**Gender**			
Female	4 (31%)	35 (31%)	0.97^*^
Male	9 (69%)	77 (69%)	
**Comorbidities**			
DM	13 (100%)	85 (76%)	0.045^#^
Chronic renal failure	1 (7.6%)	28 (25%)	0.16^*^
Hemodialysis	0 (0%)	6 (5.3%)	0.39^*^
Malignancy	1 (7.6%)	12 (10.7%)	0.73^*^
Immunodeficiency	0 (0%)	15 (13.4%)	0.16^*^
CVD (IHD, HTN, CVA, hyperlipidemia)	6 (46.0%)	61 (54.4%)	0.57^#^
**Symptoms & Signs**			
Mean duration of symptoms days (mean +/- SD)	71.58 +/-58	40.9 +/-39.8	0.015^^^
Pain	13 (100%)	88 (78.6%)	0.06^#^
Otorrhea	9 (69%)	74 (66%)	0.81^#^
Edema	9 (69%)	68 (60.7%)	0.55^#^
Cranial nerve palsy	4 (30.7%)	12 (10.7%)	0.04^*^
Granulation tissue	3 (23%)	54 (48.2%)	0.84^*^
Bilateral disease	0 (0%)	12 (10.7%)	0.21^*^
Mean duration of hospitalization - days (mean +/- SD)	20.4+/-6.5	20.7 +/-20.8	0.35^^^

SD standard deviation; DM Diabetes mellitus; CVD Cerebrovascular disease; IHD Ischemic heart disease; HTN Hypertension. ^Two-sample t-test was used for quantitative variables; Categorical variables were compared using the chi-square test^#^ or Fisher's exact test^*^ as appropriate

P-values are presented without correction for multiple comparisons, as the study is exploratory and underpowered for formal adjustment

Ceftazidime (87.1%) was the most frequently used antibacterial agent, followed by ciprofloxacin (38.7%) and meropenem (16.1%). Voriconazole (25.8%) was the most common antifungal medication, followed by fluconazole (16.1%) and itraconazole (3.2%). In both groups, the average duration of antibiotic therapy was 5 weeks (± 2). In the combined treatment group, the average duration of antifungal therapy was 5.7 weeks (± 1.8). Thirty-one patients underwent surgical intervention, primarily local debridement and mastoidectomy. Surgical intervention rates were similar between the two groups: 2 (15%) in the combined antifungal and antibiotic group and 29 (25.8%) in the antibiotic-alone group (*p* = 0.4). Only one patient received hyperbaric oxygen therapy during their initial treatment.

### Fungal culture results

Culture results were available for 121 patients, 13 from the combined treatment group and 108 from the antibiotic-only group. A total of 179 different isolates were identified, as 32 cultures yielded more than one microorganism, and multiple samples were obtained from 98 patients (ranging from 2 to 15 samples per patient).

Fungal pathogens were identified in 35 cultures (out of 179); Among these, 40% were *Aspergillus* species, 57% were *Candida* species, and one isolate was Trichophyton. *Pseudomonas aeruginosa* was the most common bacterial isolate in the antibiotic-only group (64/108, 59%), whereas it was isolated in only four cultures (30%) in the combined treatment group (*p* = 0.05).

**Fig. 2:**
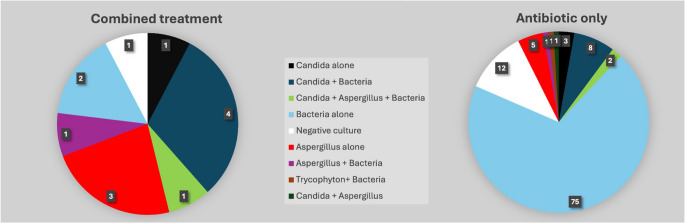
Distribution of fungal and bacterial cultures in the combined antifungal and antibiotic treatment (*n* = 13) and antibiotic-only groups (*n* = 108)

A positive fungal culture was identified in 10/13 (77%) of patients in the combined treatment group, compared to 21/108 (19%) of patients in the antibiotic-only group (*p* < 0.001). One culture was negative for any pathogen, and two grew bacteria (Staph. epidermidis and enterococci). In the combined-treatment group, microbiologists reviewed and scored all 10 positive fungal cultures with high inter-reviewer agreement (k = 0.72): two were classified as definite or highly probable fungal infection (score 1), and two, a score of 2, possible fungal NOE. All of these four cultures grew *Aspergillus* species. Six of the ten positive fungal cultures were classified as low probability of fungal NOE (score 3) and included all of the *Candida* isolations. In the antibiotic-only group, out of the 21 fungal cultures, three cultures (two *Aspergillus* and one *Candida*) were classified as definite fungal NOE. Five cultures were classified as possible (score 2), and the remaining 13 as low probability fungal NOE (score 3); 11 of these were *Candida* isolations. Nine patients in the antibiotic-only group had solely fungal growth; 6 of these were classified as definite or possible fungal NOE, yet these patients did not receive systemic antifungal treatment. The distribution of fungal and bacterial cultures in the patient cohort is depicted in Fig. 2.

### Persistent/recurrent disease

Thirty-two patients (26.4%) experienced persistent or recurrent disease, defined as treatment duration exceeding 8 weeks. This group included 6/13 patients (46%) treated with combined antifungal and antibiotic therapy and 26/112 (23%) treated with antibiotics alone (*p* = 0.07).

Of the 26 patients with persistent or recurrent disease in the antibiotic-only group, 14 continued with antibiotics alone, while 12 received additional antifungal therapy (Fig. 1). No statistically significant differences were observed in the clinical features between these two subgroups within the antibiotic-only group.

The average additional treatment duration for all patients with persistent or recurrent disease was 7.5 weeks (range 2–49 weeks) for antibiotics and 11.5 weeks (range 2–32 weeks) for antifungals. All receiving antifungal therapy also received concurrent antibiotic treatment.

A multivariate logistic regression model was constructed to predict persistent/recurrent disease, including variables that showed significant differences between the combined antifungal-antibiotic treatment group and the antibiotics-only group, such as age, DM, duration of symptoms, and cranial nerve palsy. Among these, only cranial nerve palsy remained a statistically significant predictor of persistent/recurrent disease (*p* < 0.001). Treatment with antifungal-antibiotic from the outset was not significant in this initial model (*p* = 0.538). When the model was recalculated to include all patients who received antifungal therapy at any time, both antifungal treatment and cranial nerve palsy were independently associated with persistent/recurrent disease (*p* < 0.001).

### Culture results in persistent or recurrent disease

Culture results were obtained for all 32 patients with persistent or recurrent disease. Approximately 50% (17/32) of these repeat cultures were negative, compared to 10.7% of the initial cultures obtained at the beginning of treatment.

**Fig. 3:**
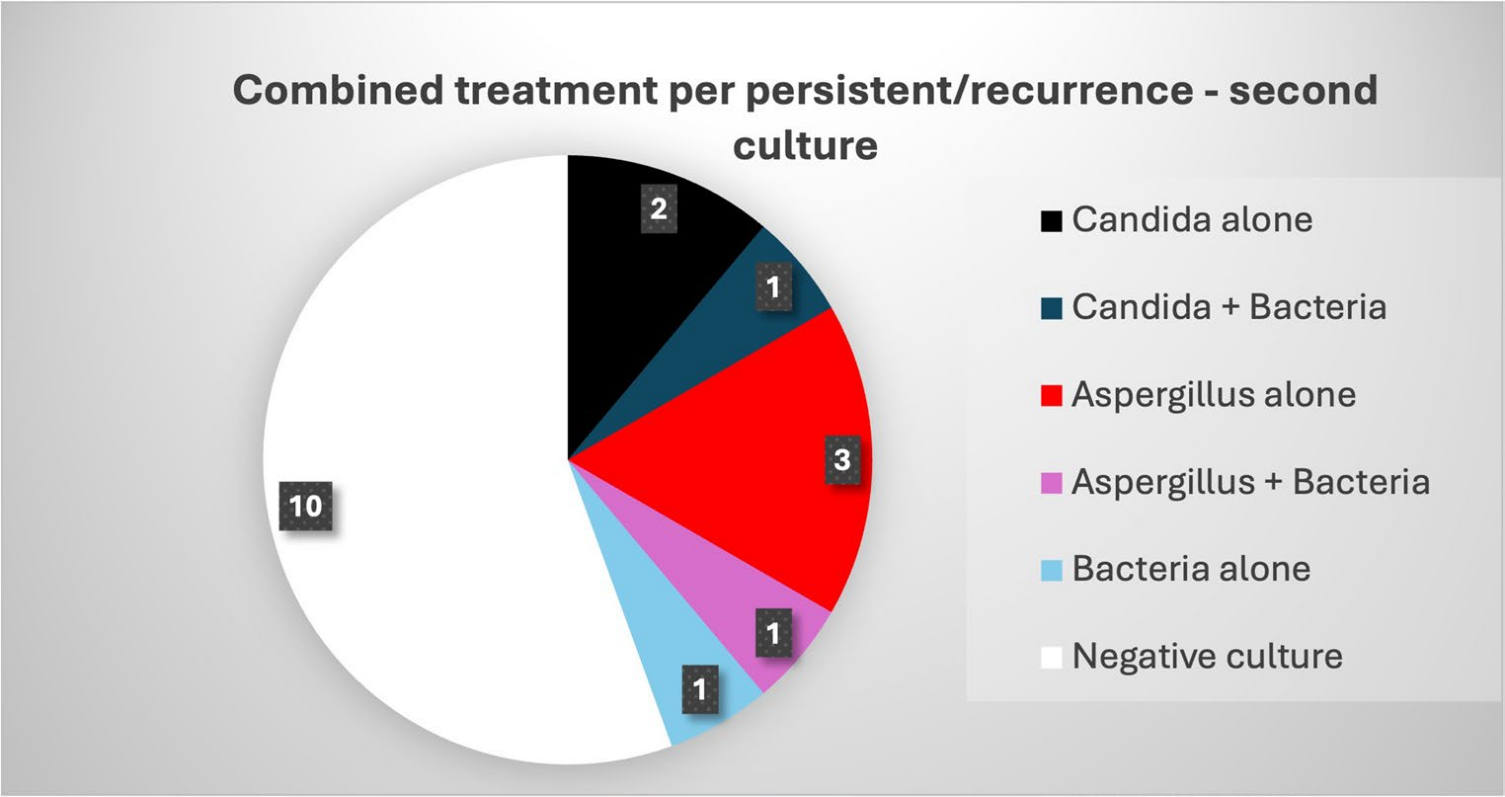
Distribution of fungal and bacterial cultures in the combined treatment per persistent/recurrence disease (*n* = 18)

In the 18 patients who received combined antifungal and antibiotic therapy for persistent or recurrent disease, fungi were identified in 7 cases (Fig. 3). Two of these cases involved new fungal isolates (both with a microbiologic score of 3- low probability) while the remaining cases involved the same fungal species as the initial culture (all with a definitive or a possible score, inter-reviewer agreement k = 0.8).

In the 14 patients who continued antibiotics alone for persistent or recurrent disease, the same bacteria were identified in the initial and repeat cultures in six patients, one patient had a positive fungal culture (*Candida* and *Aspergillus*), and the remaining seven patients had negative repeat cultures.

### Combined antifungal and antibiotic treatment vs. antibiotic alone for all the entire cohort

The clinical characteristics were re-analyzed for all 25 patients who received combined antifungal and antibiotic therapy (13 at treatment initiation + 12 after persistent/recurrence) compared to the 100 patients who received antibiotics alone. Compared to the initial analysis (Table 1), only a longer duration of symptoms (62 vs. 39 days, *p* = 0.021) remained significant. The combined treatment group exhibited significantly higher rates of cranial nerve palsy (36% vs. 7%, *p* = 0.0001), persistent/recurrent infection (72% vs. 14%, *p* < 0.000001), hyperbaric oxygen therapy (4 [16%] vs. 1 [1%], *p* = 0.0006) and surgical intervention (12 [48%] vs. 25 [25%], *p* = 0.024).

Positive fungal cultures (either initial or upon recurrence) were identified in 72% (18/25) of patients in the combined treatment group compared to only 16% (16/100) of patients in the antibiotic-only group (*p* < 0.0000001).

## Discussion

Fungal infection has been implicated in 2–20% of NOE, primarily based on case reports and small case series [9–13]. Diagnosing fungal NOE remains challenging due to the often insidious nature of the infection and the potential for fungal cultures to be misattributed to contamination. This retrospective review of NOE cases treated at two tertiary medical centers represents one of the largest cohorts studied to date. Our primary objectives were to investigate the role of fungal pathogens in NOE and to identify factors that prompted the addition of systemic antifungal therapy to the treatment regimen.

We examined the clinical features affecting the decision to initiate systemic antifungal therapy in patients with NOE. Patients in the combined antifungal and antibiotic treatment group tended to be younger, with a higher prevalence of diabetes, longer symptom duration, and higher prevalence of cranial nerve palsies, compared to those treated with antibiotics alone. These findings align with previously reported data [2, 9, 10, 13, 18]; however, none of these features uniquely or exclusively indicate fungal involvement. While 100% of patients had diabetes, so did 75% of the patients in the antibiotic-only group, implying that clinical features, though suggestive of fungal infection, are insufficient to justify antifungal therapy on their own.

Cranial nerve palsies, a well-established marker of severe NOE and a potential predictor of poor prognosis [2, 19, 20], were significantly more frequent in the combined treatment group. While some studies have linked cranial nerve palsies to fungal etiology [10, 19], others have not found such an association [20]. Our findings of 40% cranial involvement in patients treated with systemic antifungals suggest that such involvement may be a valuable clinical indicator for considering antifungal therapy. However, it is not pathognomonic for fungal infection. Importantly, cranial nerve palsies can also occur in patients with bacterial NOE and often manifest later in the disease course, limiting their utility in guiding initial treatment decisions.

The presence of a positive fungal culture alone did not consistently lead to the initiation of antifungal therapy. In the combined treatment group (*n* = 13), 76% of patients exhibited positive fungal cultures; however, three patients received antifungal therapy despite negative fungal cultures. Furthermore, eight (61%) of these patients also presented with concurrent bacterial infections (Fig. 2). Similarly, within the antibiotic-only group (*n* = 108), 21 patients demonstrated positive fungal cultures, including ten with isolated fungal growth, yet none received antifungal therapy. A systematic review by Sideris et al. reported that approximately 13% of patients with fungal NOE did not receive antifungal treatment despite positive fungal cultures [13]. This observation highlights the challenges in interpreting fungal culture results in NOE. The use of broad-spectrum antibiotics prior to culture collection can significantly impact the microbial ecology of the ear canal, potentially leading to the overgrowth of fungi and inaccurate culture results [21]. While tissue biopsies are considered the gold standard, particularly for fungal identification [22], their use is often limited in elderly and frail patients with NOE, especially when the infection involves deep, inaccessible structures within the skull base. Calcofluor white staining, a rapid technique for identifying fungal elements via fluorescence microscopy, can complement culture results by providing immediate evidence of a high fungal burden within the sample [23].

Three infectious disease specialists (HE, MK, SI) meticulously reviewed all culture results and assigned scores to assess the likelihood of fungal involvement, as described in the Methods section. We hypothesized that significant differences would be observed in the microbiological scores between the two groups, with better scores for fungal cultures in the combined antifungal and antibiotic group compared to the antibiotic-only group. However, no such significant difference was found, further emphasizing the limitations of relying solely on culture results to guide treatment decisions. Notably, the isolation of *Candida species*, especially when co-identified with bacteria, tended to receive lower reliability scores. This stems from the view of *Candida* as a common nosocomial contaminant selected by prior antibiotic treatment [24]. Despite this bias, similar proportions of *Candida* and *Aspergillus* species were observed in both treatment groups, consistent with findings reported in the literature for other fungal NOE cases [13, 18]. 

Half of the patients in the combined treatment group experienced a prolonged disease course requiring treatment beyond the accepted 8 weeks, compared to 23% of patients in the antibiotic-only group. Importantly, in 12 out of 26 patients in the antibiotic-only group who ultimately required systemic antifungals, new fungal growth was identified in only two patients. This observation supports the hypothesis proposed by Hamzani et al. [10], suggesting that fungal infections arising late in the course of antibiotic treatment may represent new opportunistic infections rather than previously undetected pathogens. Given that we excluded patients who died during treatment from our analysis, the need for prolonged treatment can serve as an indicator of disease aggressiveness. Notably, disease-specific mortality rates were similar between the groups (7% and 9%), comparable to the 5% mortality rate reported in other NOE studies [25] and the 4% explicitly reported for fungal NOE [13]. 

However, analysis of the entire cohort demonstrates that patients who received antifungal therapy, whether initiated concurrently with antibiotics or added later, generally experienced more aggressive disease courses. These patients exhibited a significantly higher rate of prolonged treatment, often requiring an additional 3–4 months of therapy beyond the typical six-to-eight-week course. This finding aligns with the observation that other forms of fungal osteomyelitis frequently require prolonged treatment regimens [26]. 

This study leverages a large cohort of NOE cases collected over three decades from two tertiary centers, providing a valuable dataset for investigating the role of fungal infections in this challenging disease. Nevertheless, it is still a relatively small cohort, exploratory in nature and involved multiple comparisons and several underpowered subgroup analyses, which may affect our conclusions. The exclusion of patients who died during treatment may introduce survivorship bias. However, as deaths were few and occurred in both groups, their impact was likely minimal. The retrospective nature of the study presents inherent limitations. Variations in diagnostic approaches, treatment protocols, and documentation practices across time and between physicians may have introduced biases and hindered the collection of complete and consistent data. Conducting a prospective, single-center trial in this rare and often prolonged disease would be challenging and may not be feasible. While this study did not definitively establish strict indications for initiating antifungal therapy, it highlights the critical need for a comprehensive, universally adopted, and multidisciplinary treatment protocol for NOE to improve diagnostic accuracy, optimize treatment decisions, and ultimately improve patient outcomes.

**Fig. 4:**
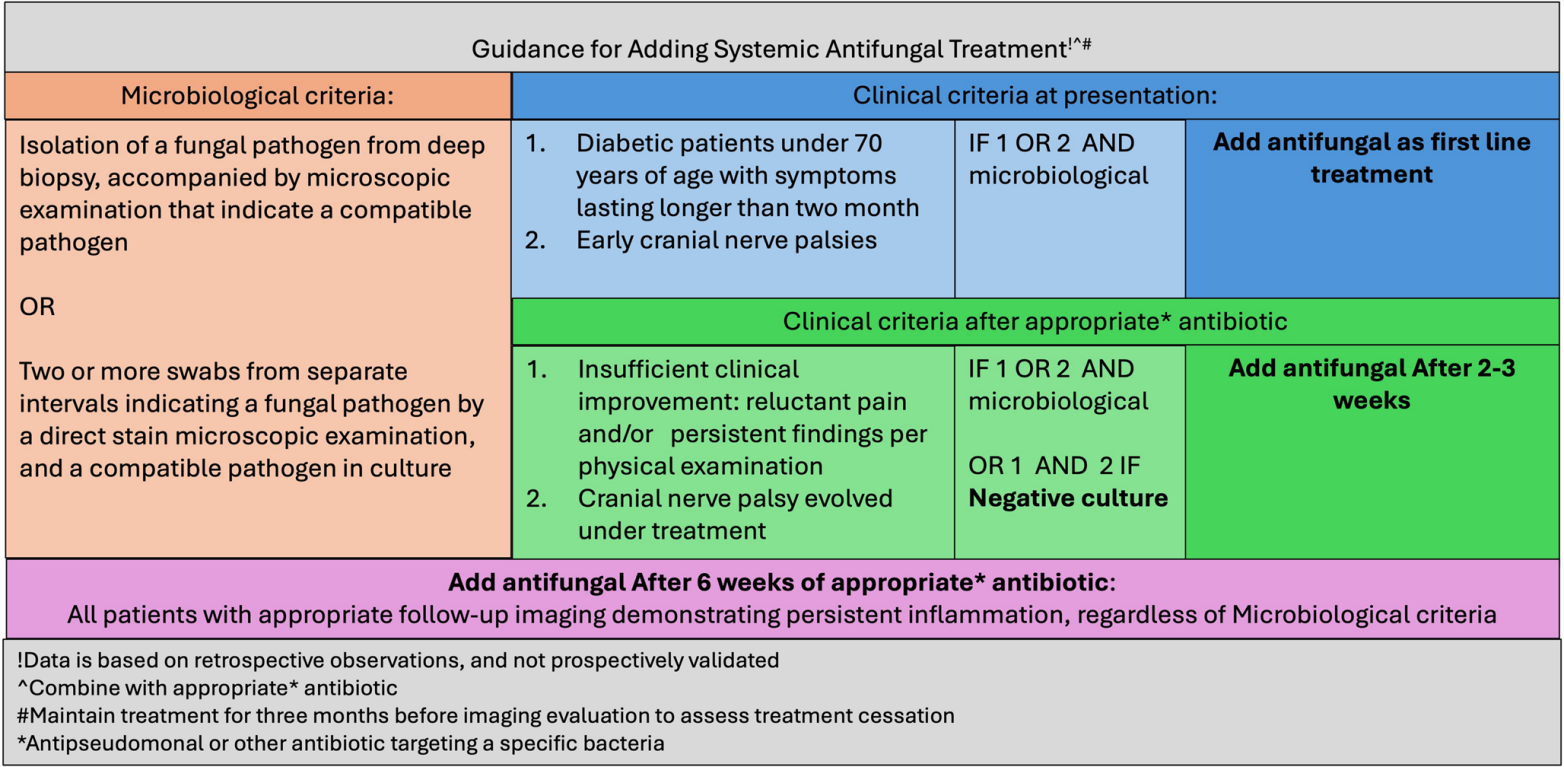
Guidance for adding systemic antifungal treatment, based on retrospective observation

We suggest defining the diagnosis based on clinical and microbiological criteria as presented in Fig. 4.

Managing these complex patients requires a multidisciplinary approach, which often results in a conflict between the otolaryngologist and the infectious disease specialist. Widespread implementation will not only facilitate standardized care but also enable prospective evaluation of the protocol’s effectiveness and its impact on patient outcomes in the coming years.

## Conclusion

This study underscores the diagnostic and therapeutic challenges associated with fungal NOE.

While clinical factors such as cranial nerve palsy, prolonged symptoms, and diabetes, along with positive fungal cultures, were associated with the use of antifungal therapy, none of these factors alone provided definite indications for its initiation. Notably, patients receiving systemic antifungal therapy exhibited higher rates of persistent disease, requiring prolonged therapy. Further prospective research is warranted to refine diagnostic criteria, optimize treatment protocols, and identify patient subgroups that may benefit most from the addition of antifungal therapy. A multidisciplinary approach involving otolaryngologists, infectious disease specialists, and microbiologists is crucial for improving the management of this challenging condition.

## Data Availability

Data collected for this study can be made available by the corresponding author upon request.
